# The Need for an Ombudsman in the Indian Medical Education and Health System: A Medical Teacher’s Perspective

**DOI:** 10.7759/cureus.85316

**Published:** 2025-06-04

**Authors:** Venkataramana Kandi

**Affiliations:** 1 Clinical Microbiology, Prathima Institute of Medical Sciences, Karimnagar, IND

**Keywords:** educational environment, government of india, medical education, national medical commission (nmc), ombudsman, quality

## Abstract

The Government of India's (GOI) initiatives to increase the number of Bachelor of Medicine and Bachelor of Surgery (MBBS) graduates by establishing more and more medical colleges have been showing their repercussions. There are several doubts over the quality of medical education (ME) and the competency of MBBS graduates. In addition, the National Medical Commission (NMC), the body that regulates ME and medical practice in India, has been busy ensuring the uniformity of ME standards by conducting regular inspections. The NMC ensures infrastructure, faculty, and patient availability before permitting colleges to admit students. Several issues are plaguing faculty, students, and other personnel working in medical colleges, which could potentially influence the educational environment (EE) and the competency of graduating students. Evidence of disparity in infrastructure, faculty, and EE in medical colleges confirms the necessity of holistic improvements. Therefore, it is suggested that the NMC utilize ombudsman services in medical institutions, which have been successfully implemented in the banking and insurance sectors.

## Editorial

The standard of medical education (ME) in India has been significantly impacted by the exorbitant increase in medical colleges and the rise in students pursuing medical degrees. The functioning of these colleges is severely affected by the unavailability of qualified teachers and a lack of adequate infrastructure. Medical students gain admission into Bachelor of Medicine and Bachelor of Surgery (MBBS) programs following stiff competition and extreme hard work. The educational environment (EE) in medical colleges is less than satisfactory, especially owing to complacency on the part of college managements, including both government and private institutions. While the establishment of medical colleges across the nation seems to have been a priority for the current Indian government, the quality of ME and graduating students has been greatly influenced by the lack of oversight of these institutions.

The National Medical Commission (NMC), which is the regulatory body for ME and the health system in India, has been complacent in permitting admissions to some medical colleges operating in temporary establishments with minimal infrastructure. This puts excessive pressure on students pursuing graduate degrees in such colleges. Moreover, faculty recruitment in medical colleges does not follow any defined criteria, except for the possession of the desired qualifications [1]. Additionally, the NMC does not prescribe a pay scale, as is done by the University Grants Commission (UGC), which regulates higher education in India. This results in a disparity of faculty salaries, creating instability and forcing frequent faculty movement from one college to another in search of salary hikes. Furthermore, many colleges have ghost faculty who are listed only on paper, attend NMC inspections, but do not regularly participate in academic activities. This affects both theoretical and practical training, compromising students’ ability to comprehend subjects.

The faculty deficiencies can be attributed to a lack of interest among MBBS graduates in pursuing pre- and para-medical subject specializations, including anatomy, physiology, biochemistry, microbiology, pharmacology, and pathology. Only the pathology subject has some preference among MBBS graduates, especially owing to earning opportunities related to its specialization. Deficiencies in these subjects can potentially affect students' learning abilities. Considering these subjects are the foundation for future clinical training, improper training can severely affect the skill development and competency of medical graduates. Another interesting fact is that the available faculties in the pre- and para-subjects lack interest in teaching, negatively reflecting on the teaching and learning (TL) process. The teachers in these subjects have been involved in an exam-oriented TL approach, which significantly affects students’ mindsets, limiting their orientation to exam performance and affecting their competencies and skill-acquiring abilities. In some medical colleges, the pre- and para-subject faculties are utilized to teach other allied medical sciences undergraduate students, including dental sciences, nursing, laboratory technology, and biomedical sciences. Faculties utilized for other courses deprive medical students of their attention, affecting their academic activities. The student-to-faculty ratio is also affected by the high admission numbers, as evidenced by some colleges being permitted 250 or more students in one batch year. This creates crowding during practical training, limiting students' learning capabilities [2].

Similar problems affect students' clinical training, mostly due to overcrowding, lack of patients, and inadequate infrastructure. Since most medical colleges are established in rural areas, the lack of awareness among people leads to negative beliefs regarding seeking medical care. Furthermore, faculty members in the clinical departments have private practices, limiting their availability for academic activities. Despite government directives and college management's vigilance, many clinical departments are deprived of full-time faculty, affecting students' TL activities [3].

These circumstances severely affect the students, leaving them stressed and helpless. The college management does not have a system to get student feedback and make necessary amendments. Due to an overwhelming rise in medical colleges in the last decade, the NMC appears clueless and unresponsive about issues plaguing ME. The primary issues pointed out by the students include teachers concentrating more on note-giving, in addition to unilateral and monotonous classroom lectures. The teachers give assignments neither by providing constructive feedback nor by assessing them appropriately, but marks are awarded based on students’ behaviour. Students also felt the amenities provided in the hostel were less than satisfactory, forcing them to leave the hostel and the college campus to live in rented accommodation. This puts additional pressure on students concerning travelling, expenses, security, and time limitations, reducing their concentration on studies. Furthermore, there is emerging evidence of a rise in psychological problems and deaths due to suicide among medical students.

Teachers’ perspectives reveal exploitation by the college management, as they are not provided with regular salary increments, and there is no uniformity in faculty pay. Additionally, teacher recruitment in most colleges is carried out temporarily, based on yearly contracts. Teachers working in some government and private medical colleges are not paid regular salaries, leaving them unhappy and looking for a change or transfer to a better college. These uncertainties among teachers reflect on their mindsets, making them complacent in carrying out their duties. Furthermore, teachers fear for their jobs and fail to point out the college’s deficiencies to the administration and the regulatory bodies.

Given the rapid growth of medical schools and the mounting worries about the deteriorating quality of ME, the NMC must take the initiative to solve the issues that could affect the current and future state of India's medical and health system. To solve the problems affecting the Indian medical and health systems, it is recommended that the NMC designate an ombudsman at the institutional, state, and national levels. Ombudsmen are already playing a key role in the Indian banking and insurance sectors.

The purpose of an ombudsman is to investigate and resolve complaints against public and private entities, often to identify and address systemic issues. Mediating disputes or making recommendations, ombudsmen typically act as an unbiased third party. An ombudsman may work domestically and internationally, within an organization or business. Ombudsmen are neutral in their operations, and their main duties include addressing complaints from individuals or groups regarding poor service, unfair treatment, or violations of their rights.

Ombudsmen can offer informal, fair, and confidential grievance redressal procedures for students, employees, and administrators. Their education offices seek to ensure adherence to policies and promote communication while facilitating just and equitable responses to college-related concerns. A prior study highlighted the importance of ombudsmen in higher education and found that many constituents find their services help personalize institutions. By having an ombudsman, the institution demonstrates its concern for its people and acknowledges the importance of offering informal dispute resolutions to college students and other members [4].

**Figure 1:**
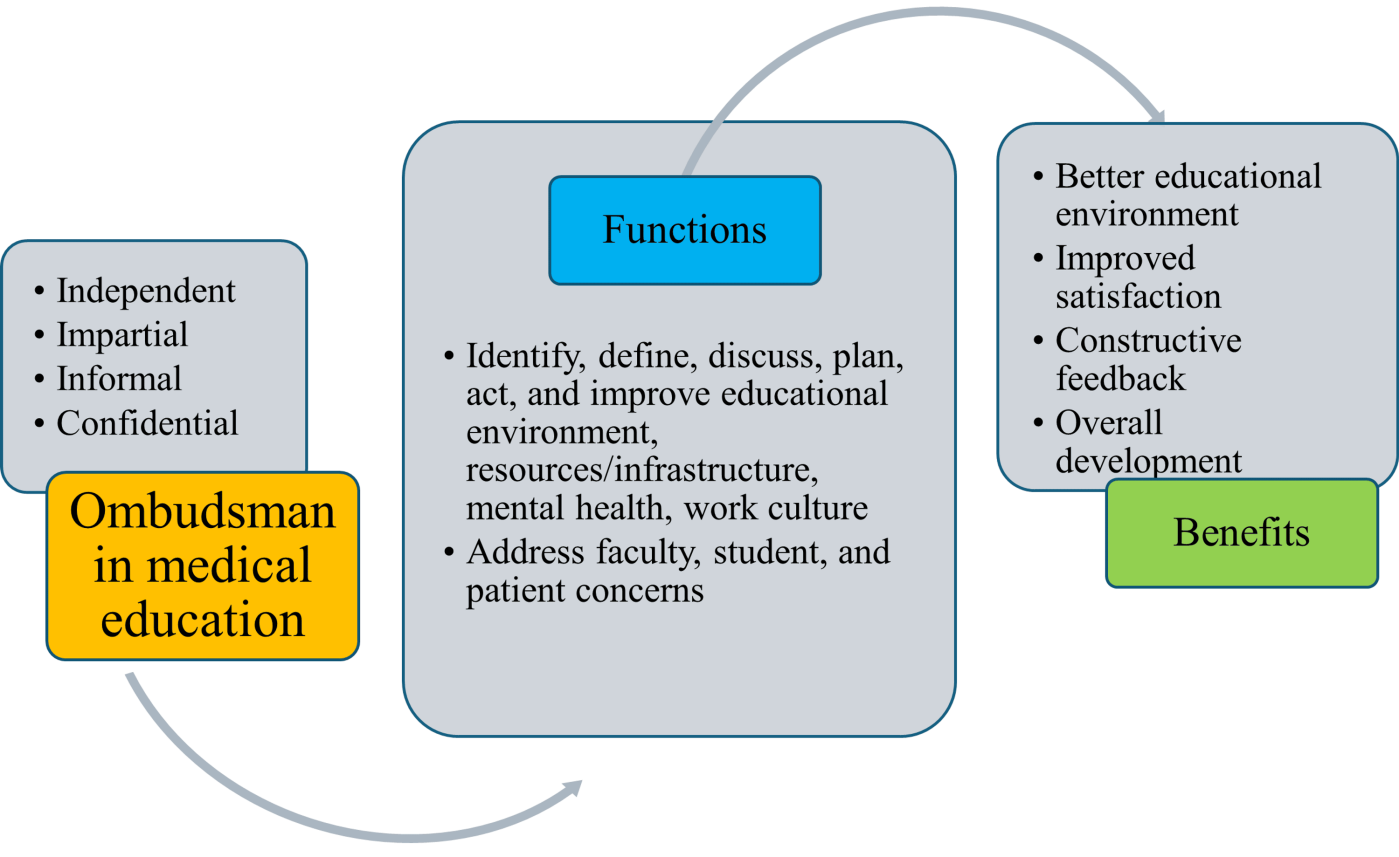
Characteristics, functions, and benefits of ombudsman in medical education Image credit: Venkataramana Kandi

The selection process, escalation pathways, and examples of proactive concerns being addressed have all been described in recent observations about the role of ombudsmen in American graduate ME. Independence, impartiality, informality, and confidentiality are the four essential components of an ombudsman's operation. The ombudsmen's escalation pathways include identifying the problem, defining it, discussing it, planning, acting on it, and improving it by coming up with solutions. The reporting categories addressed by an ombudsman include issues regarding professionalism (faculty, students, and patients); education and training gaps (EE and TL materials); resources and service requirements (duty hours and facilities like rest rooms and food); mental health and wellness (personal and campus environment); and culture and morale (social life, appreciation, and psychological safety). Confidential reporting of unethical activity significantly improved between the pre-ombudsman and post-ombudsman periods. More people were coming out with concerns without worrying about intimidation or reprisals. Additionally, greater satisfaction with the process of handling issues and complaints confidentially is a reflection of the post-ombudsman atmosphere (Figure 1) [5].

In conclusion, the current phase of ME and the health system in India is fragile, owing to massive expansion. Ensuring the quality of ME is paramount from a public health perspective. Therefore, the NMC is suggested to utilize ombudsmen to identify the issues and implement corrective measures to improve the quality of ME and the standards of Indian medical graduates. 
